# Water-Soluble Epoxy Resins as an Innovative Method of Protecting Concrete Against Sulfate Corrosion

**DOI:** 10.3390/ma19020364

**Published:** 2026-01-16

**Authors:** Wojciech Kostrzewski, Ireneusz Laks, Marta Sybis

**Affiliations:** Department of Construction and Geoengineering, Poznan University of Life Sciences, Piątkowska 94 E, 60-649 Poznan, Poland; wojciech.kostrzewski@up.poznan.pl (W.K.); ireneusz.laks@up.poznan.pl (I.L.)

**Keywords:** sulfate corrosion, polymer-modified concrete, water-based epoxy resins, concrete durability, sewer infrastructure protection

## Abstract

Sulfate corrosion is a significant durability issue for concrete used in sewage and hydraulic infrastructure. In sulfate-rich environments, the formation of expansive products (e.g., ettringite and thaumasite) leads to a progressive loss of performance. Unlike conventional protection methods, which rely on surface-applied coatings or impregnation, this study examines the use of water-dilutable epoxy resins as an internal, volume-wide admixture dispersed throughout the concrete matrix to provide whole-body protection. The experimental program evaluated the mechanical performance, microstructure, and sulfate ion ingress/penetration dynamics of resin-modified concretes. The results suggest that using the appropriate amount of resin can limit the penetration of aggressive ions and slow the harmful changes associated with sulfate attack while maintaining the material’s overall performance. Overall, these findings suggest that water-based epoxy admixtures are a promising strategy for improving the durability of concrete in sulfate-exposed environments. They also provide guidance for designing more resistant cementitious materials for modern infrastructure applications.

## 1. Introduction

Concrete is one of the most important building materials, widely used in various fields of engineering, including building construction, hydrotechnical engineering, and transport infrastructure. Due to its durability and resistance to mechanical loads, concrete seems to be an ideal material for use in demanding operating conditions. However, despite its many advantages, the durability of concrete structures is seriously threatened by various corrosive factors, including chemical, physical, and biological effects [1,2]. Concrete corrosion, defined as the gradual degradation of its structure as a result of aggressive environmental factors, poses a significant challenge for modern materials technology [3,4].

Among the most common forms of concrete corrosion are chemical corrosion, including sulfate, chloride, and carbonate corrosion. Sulfate corrosion, which is particularly dangerous in sewage and hydrotechnical environments, is the result of a reaction between sulfates contained in groundwater, sewage, and the atmosphere with calcium hydroxide present in cement, leading to the formation of expansive reaction products such as ettringite and thaumasite [5,6,7]. This process results in the degradation of the concrete microstructure, increased porosity, and weakened mechanical properties, which in turn leads to serious structural damage [8,9,10].

Concrete corrosion can also be caused by organic and inorganic acids, leading to the decomposition of cementitious components and weakening of the concrete structure [11,12,13]. In the case of sewage infrastructure, biocorrosion processes are particularly problematic. These result from the presence of sulfur bacteria “Thiobacillus,” which oxidize hydrogen sulfide into sulfuric acid, significantly lowering the pH of the concrete environment and accelerating its degradation [10,14,15]. Biocorrosion of concrete in sewage conditions leads to a significant loss of structural strength and requires costly repair measures [16,17].

The literature emphasizes that the appropriate selection of concrete composition, including the use of cements with increased corrosion resistance, chemical admixtures, and surface protection methods, can significantly reduce the risk of material degradation [18,19]. In particular, modifications to concrete using polymers and protective coatings, such as epoxy resins, increase the resistance of concrete to aggressive environments [20,21]. The introduction of polymers into concrete has a positive effect on its microstructure, reducing porosity and improving barrier properties [22,23,24]. Particular attention is paid to the use of polymer admixtures, such as styrene-butadiene latexes or acrylic resins, which increase the resistance of concrete to chemical aggression [25,26]. Although surface-applied protective systems (coatings, linings, impregnation) are widely used to limit the ingress of aggressive agents, their long-term effectiveness can be compromised in practice by damage and loss of continuity of the barrier layer. Reported failure modes include partial loss of adhesion (e.g., moisture/osmotic-pressure-related blistering), microcracking caused by coating shrinkage or cracking of the concrete substrate, and peeling/delamination initiated by the penetration of aggressive substances through local defects [27,28]. Such vulnerabilities are particularly relevant in aggressive service environments (e.g., wastewater infrastructure), where field observations have documented large-area losses of epoxy protective coatings and rapid progression of concrete deterioration within a short service period [29]. Recent reviews also emphasize that surface treatments often require periodic renewal and may be exposed to mechanical wear in service (e.g., high-traffic or operational conditions), further motivating complementary strategies that are less dependent on an intact surface film [30].

In recent years, new technologies for protecting concrete against corrosion have emerged, including the use of geopolymers, hydrophobic coatings, and corrosion inhibitors [31,32,33]. Geopolymers, which are an alternative to traditional cements, are highly resistant to chemical corrosion and can be used in aggressive environments [34,35]. Superhydrophobic coatings, on the other hand, prevent moisture adsorption and reduce the rate of penetration of aggressive agents into the interior of the concrete [32].

The development of new technologies for protecting concrete against corrosion, including innovative polymer and composite materials, is an important area of research in materials engineering. Optimization of concrete composition and modern protection methods allows for a significant extension of the durability of structures and a reduction in operating costs. Future research should focus on further improving the barrier properties of concrete, including the use of nanomaterials and advanced methods of corrosion process diagnostics [29]. The development of water-based epoxy resins as concrete protection agents opens up new perspectives in terms of the durability of concrete infrastructure, especially in conditions of intense exposure to aggressive chemical environments [22,36,37].

The use of these materials allows for a reduction in the rate of concrete structure degradation, especially in conditions of exposure to sulfate corrosion. The results obtained indicate that the use of appropriate concentrations of epoxy resin leads to a significant reduction in the intensity of corrosion processes, which translates into an extension of the durability of infrastructure elements. The advantage of this method is the possibility of protecting the entire volume of concrete, which is an important supplement to classic surface methods, such as impregnation or protective coatings. In view of the growing requirements for the durability of building materials and the need to reduce operating costs, the use of this approach may be a valuable direction for the further development of concrete technology used in environments with a high degree of chemical aggressiveness.

## 2. Materials and Methods

### 2.1. Materials

Portland cement CEM I 42.5 R manufactured by Heidelberg Materials Polska (Poznań, Poland) was used to conduct tests on the resistance of concrete to sulfate corrosion. This cement was chosen due to its chemical composition, which makes it more susceptible to aggressive environments compared to sulfate-resistant cements, allowing for faster recording of corrosion effects within a limited testing period. CEM I 42.5 R is characterized by high strength and rapid strength gain, which allows for testing in a shorter period of time.

A mixture of 0–2 mm quartz sand and 2–8 mm basalt grit, supplied by Kruszywa Polskie Sp. z o.o. (Małogoskie, Poland) was used as an aggregate. These aggregates were selected for their high mechanical resistance and low susceptibility to chemical reactions. The water used in the mixture came from the water supply system and met the requirements of the PN-EN 1008 standard [38].

In addition, in order to increase the resistance of concrete to aggressive sulfate factors, selected samples were modified with MasterTop 686 water-based epoxy resin manufactured by Master Builders Solutions (Oosterhout, The Netherlands). This resin was used in various concentrations: 5%, 10%, and 20% by weight of cement. This made it possible to assess dose-dependent effects and to identify the most promising resin dosage(s) for improved resistance to sulfate corrosion within the scope of the present testing program. Vinapor from Sika (Baar, Switzerland) was used as a defoaming admixture.

### 2.2. Test Methodology

#### 2.2.1. Preparation of Concrete Cubes

The preparation of test samples was a key stage in ensuring the repeatability and reliability of the results. First, the components of the concrete mix were measured according to the recipes shown in Table 1, taking into account different concentrations of epoxy resin.

Because the epoxy resin was supplied as a water-dilutable aqueous dispersion, the “water” value reported in Table 1 refers only to the added mixing water. The resin dispersion contained approximately 59% water and 41% resin solids (by mass). Therefore, as the resin dosage increased, the added mixing water was reduced to compensate for the water introduced with the resin dispersion. The apparent decrease in “water” from W-0 to W-20 thus reflects a redistribution between added water and resin-borne water, rather than a reduction in the total water present in the mixture. The ingredients were thoroughly mixed in a laboratory mixer with forced mixing for 3 min. The concrete mixture was then placed in 15 × 15 × 15 cm molds. To ensure adequate compactability of the mixture, the samples were compacted on a vibrating table for 30 s. After forming, the samples were covered with foil to protect them from excessive water evaporation. After 24 h, the molds were removed and the samples were cured. The curing process consisted of 5 days of aging in tap water, followed by storage in a laboratory under controlled humidity conditions (50–65%) until day 28. After the aging process was completed, the samples were tested for resistance to sulfate corrosion in accordance with the accepted methodology.

#### 2.2.2. Experimental Research

The research aimed to confirm the effectiveness of epoxy resins as a means of limiting the progression of sulfate corrosion.

The research was divided into two stages:

- Long-term tests (2 years) covering two variants of concrete samples in large numbers (36 samples per variant).
- Short-term tests covering five variants of standard mortars mixed with epoxy resin, where each series consisted of two samples.

In both stages, corrosion progress was monitored by measuring the weight loss of the samples. The procedure consisted of drying the samples, carefully cleaning them of the corroded mortar layer, and then weighing them accurately to assess weight loss. The results were analyzed in the context of the effect of different epoxy resin concentrations on corrosion reduction.

Stage I: Long-term research

The tests involved placing concrete samples measuring 7 × 7 × 7 cm, cut from 15 × 15 × 15 cm samples. This sample size was chosen due to the limited space available in the laboratory. For the same reason, the tests were limited to two types of concrete. Samples of reference concrete (marked with the symbol W-0) without admixture and samples with 5% resin content (marked with the symbol W-5) were selected for testing. A 5% admixture of resin to the cement mass is the maximum amount of the agent that can be used as an admixture. In parallel, higher resin dosages (10% and 20%) were examined in the short-term stage to establish the dose–response trend and to identify candidate formulation(s) with the most favorable performance for subsequent long-term validation. Within the present manuscript, the two-year exposure dataset is therefore limited to the 0% and 5% variants. Thirty-six samples were taken for each variant.

The samples were placed in tanks in two separate layers: reference samples W-0 and samples containing resin W-5. The tanks were filled with an aqueous solution of sulfuric acid with a pH of 2. This acidity is recommended/commonly adopted in laboratory studies, including [39], and it is widely used as a severe, accelerated condition to represent the acidic stage of sewer-related deterioration, where sulfuric acid (often biogenic in real systems) is produced as a consequence of H_2_S oxidation and can lead to a strong local decrease in surface pH [8,9,10,14]. Maintaining a controlled pH provides a reproducible chemical driving force and enables efficient comparison of mixtures under highly aggressive acid exposure; similar approaches have been used in test procedures developed to investigate biogenic sulfuric acid corrosion and to compare material performance under defined conditions. It should be noted that a purely chemical H_2_SO_4_ solution does not reproduce the full biological complexity of sewer environments (e.g., biofilm development and biological kinetics); therefore, the outcomes should be interpreted primarily as comparative performance under strong acid attack rather than as a direct prediction of in-service deterioration [9,10,40]. The solution was checked periodically (once a week) and replaced if necessary. The progress of corrosion was measured by monitoring changes (losses) in the mass of the samples. This involved periodically removing samples from the solution, drying them to a constant mass, and brushing them to remove loose corrosion products. The samples were then weighed. After weighing, the samples were returned to the tank. Standardized cleaning procedure prior to weighing: After removal from the corrosion medium, the specimens were cleaned manually using a stainless-steel wire brush (10 × 3 cm). Brushing was performed over all six faces of each 7 × 7 × 7 cm specimen under a controlled nominal load of approximately 5 kg. The brushing duration was governed by an end-point criterion rather than a fixed time: the procedure was stopped when no further loose corrosion products were observed to detach onto a clean white paper sheet placed beneath the specimen. After cleaning, the specimen was weighed, and the detached corrosion products collected on the paper were also weighed.

It was assumed that the first measurement of mass loss would be taken after one year, and the second after two years. Both the number of samples and the duration of the experiment should rule out the occurrence of a random effect that could lead to incorrect conclusions.

Stage II: Short-term research

In the second stage, after the completion of long-term tests, short-term tests were conducted, which lasted 6 months and included five variants of cement mortar beams with the addition of epoxy resin:

- W-0: samples without resin additive,
- W-5: samples with 5% resin content,
- W-10: samples with 10% resin content,
- W-20: samples with 20% resin content.

The choice of standard mortars mixed with epoxy resin instead of concrete samples was based on the assumption that the corrosion process would proceed faster for mortars. The results obtained in a relatively short time will enable an objective assessment of the impact of the percentage content of resins on the corrosion process.

After 6 months, mass loss measurements were performed, and chemical and surface analyses of the samples were carried out. The purpose of these analyses was to quantitatively and qualitatively determine the impact of the percentage of epoxy resin on the progress of sulfate corrosion.

Both stages of the research provided valuable data that can be used to further optimize the composition of epoxy resin-reinforced cement mixtures, especially in the context of applications in environments exposed to sulfate corrosion.

## 3. Results

### 3.1. Long-Term Studies—Analysis of Weight Loss

The raw initial-weight data are provided in Supplementary Materials Table S1. A total of n = 36 specimens were tested per variant (0% and 5%). The statistical analysis of the experiment results was divided into two main stages:

- preliminary analysis, the purpose of which was to check whether the initial masses of the samples for both variants did not differ significantly statistically,
- proper analysis, the purpose of which was to check whether the mass losses in both samples differ significantly statistically, and whether the corrosion process over time does not proceed equally for both variants.

**Figure 1:**
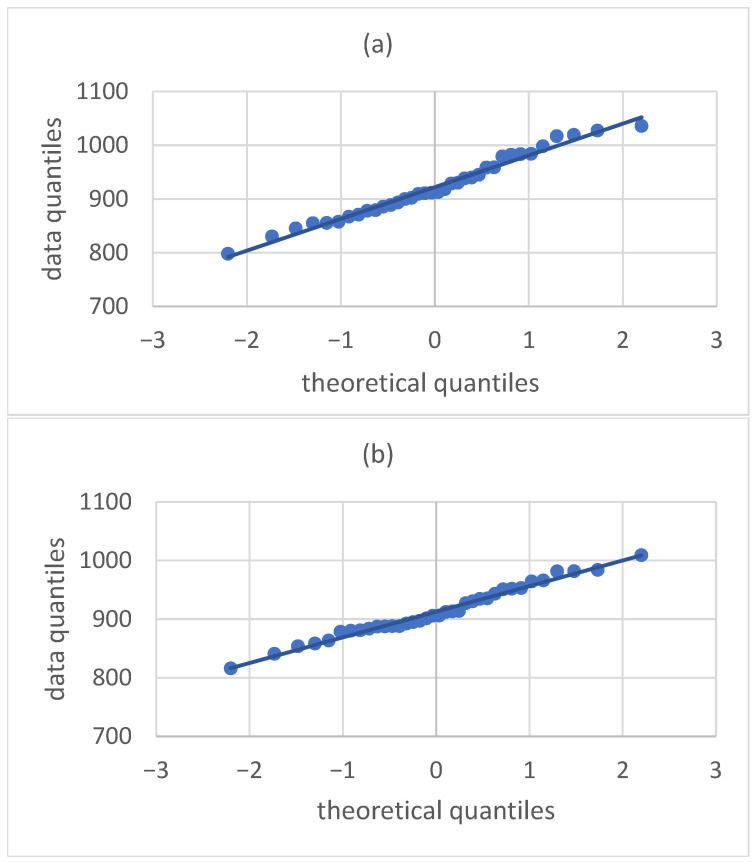
Q-Q (quantile-quantile) plots of sample weights for (**a**) Q-Q chart for the 0% variant and (**b**) Q-Q chart for the 5% variant.

Preliminary analysis stages:

1. Descriptive statistics were calculated (mean, max, min, standard deviation). Summary in Table 2.
2. Verification of Student’s *t*-test assumptions:
  The Shapiro–Wilk test and Q-Q (quantile-quantile) plots were performed to verify whether the data distribution for both variants is consistent with the normal distribution. The results are presented in Table 3 and the Q-Q plots in Figure 1.
3. The Levene test was performed at a significance level of α = 0.05 to test the null hypothesis of homogeneity of variance in the samples (Table 4).
4. Verification of whether the average weights [g] of the samples differ statistically significantly (Student’s *t*-test) (Table 5).

Samples must be made with sufficient accuracy to enable comparison of mass losses, which will be significantly smaller than their mass. If the two series are not comparable, it is even more impossible to compare mass losses.

Stages of the actual analysis:

1. Checking whether there are outliers in the seriess containing percentage weight losses—Tukey’s Fences test. Outliers are marked in red in Table S3 Supplementary Materials.
2. Checking whether the samples are normally distributed—Shapiro–Wilk test. Results in Table 6.
3. Analysis of statistically significant differences between individual series—one-way analysis of variance (one-way ANOVA) and Tukey’s post hoc test (Table 7 and Table 8).

The demonstration of statistically significant differences between individual series will indicate different patterns of sulfate corrosion in both variants. Tukey’s post hoc test allowed for the identification of pairs of samples that are statistically different.

I. Preliminary analysis

**Table 2:** Descriptive statistics of the starting weight of samples for the 0% and 5% variants.

Statistics (a)	0% Option	5% Option
Average [g]	922.05	913.02
Max [g]	1035.85	1024.92
Min [g]	798.21	790.66
Standard deviation [g]	59.39	58.84

**Table 3:** Shapiro–Wilk test parameters for the starting mass of samples for the 0% and 5% variants.

Parameter	0% Option	5% Option
*p*-value	0.5197	0.5292
W	0.9732	0.9735

Shapiro-Wilk test conclusion.

The data distributions in both samples (variants) have the characteristics of a normal distribution, as confirmed by both the high *p*-values significantly exceeding the assumed significance level of α = 0.05 (Table 3) and the arrangement of points on the Q-Q plot, which are arranged along a straight line. Therefore, there is no basis for rejecting the null hypothesis H0, which assumes that the data are normally distributed in the variants studied.

Levene’s test—conclusion.

The *p*-value for Levene’s test (Table 4) is 0.09388 and is greater than 0.05. Therefore, there are no grounds for rejecting the null hypothesis. This means that at a significance level of 0.05, we cannot conclude that the variances in both measurement series are significantly different. Furthermore, the F-statistic value falls within the acceptance range.

**Table 4:** Levene test results for a series of mass measurements for the 0% and 5% variants.

F	Statistical Acceptance Range F	*p*-Value
2.884	[0:3.9778]	0.094

To confirm or reject the H0 hypothesis of no significant difference between the variants under study, a two-sample Student’s *t*-test was performed (under the assumptions) at a significance level of α = 0.05. The results of the test are presented in Table 5.

Student’s *t*-test—conclusion.

The values presented in Table 5 indicate that the null hypothesis H0 about the absence of differences between the two data sets cannot be rejected. This is confirmed by the high *p*-value and the T-statistic, and the difference in means falling within the acceptance ranges.

**Table 5:** Results of the two-sample Student’s *t*-test for weight measurements for the 0% and 5% variants.

*p*-Value	T	Statistical Acceptance Range T	Difference in Averages	Area of Acceptance of the Difference in Means
0.4444	0.7691	[−1.99:1.99]	9.47	[−24.55:24.55]

II. Proper analysis

During the experiment, two measurements of the weight loss of the sample set were taken. Raw data for sample weight loss measurements for both variants are provided in Tables S2 and S3, Supplementary Materials.

Checking for outliers—Tukey’s Fences test—conclusion.

Comparative analyses were performed on relative percentage values of weight loss. Tukey’s Fences test was performed to determine outliers for each series of percentage weight loss. For the 0% and 5% variants, outliers occurred for the series from 1 March 2022 (2 values marked in red in Table S3, Suplementary Materials). These values were removed from further comparative analyses.

Verification of assumptions about the normal distribution of weight loss data—conclusion.

The Shapiro–Wilk test was also performed for individual series, and the summary results are presented in Table 6. All series are consistent with the normal distribution, which is confirmed primarily by high *p*-values significantly exceeding the assumed significance level of α = 0.05.

**Table 6:** Shapiro–Wilk test parameters for percentage weight loss for variants with 0% and 5% resin content.

Test Parameter	0% (1 March 2022)	0% (27 March 2023)	5% (1 March 2022)	5% (27 March 2023)
*p*-value	0.9382	0.9651	0.6138	0.09339
W	0.9863	0.9885	0.9751	0.9484
Count (n)	34	36	34	36
$\mathrm{Mean}\;(\bar{x}$)	0.941	1.6917	0.9307	0.9062
Median	0.942	1.7425	0.9055	0.898
Sample standard deviation (s)	0.1738	0.3677	0.1558	0.4333
Skewness coefficient S	0.01027	−0.2796	0.514	0.3026
Kurtosis K	0.4426	0.0899	0.77	−0.9389

ANOVA test assumptions.

The null hypothesis of the one-way ANOVA variance test assumes that the mean values in all groups are equal. The significance level of the test was α = 0.05. For ease of analysis, the following measurement series designations were adopted:

- x1—series for the 0% variant from 2022.
- x2—series for the 0% variant from 2023.
- x3—series for the 5% variant from 2022.
- x4—series for the 5% variant from 2023.

ANOVA test conclusion.

The calculated *p*-value is 3.33 × 10^−16^ and strongly supports the alternative hypothesis (H_1_), i.e., that the means in some series differ significantly. The F statistic is 54.38, which is significantly higher than the critical value (2.6712) at a significance level of 0.05. This means that the test result is outside the acceptance region of the null hypothesis, which strengthens the conclusion to reject it. The η^2^ index is 0.55, which means that 55% of the variability in the data can be attributed to differences between groups.

The results of the post hoc Tukey HSD/Tukey–Kramer test (Table 7 and Table 8) indicate that there are statistically significant differences between the following pairs of groups:

- x1–x2.
- x2–x3.
- x2–x4.

**Table 7:** Results of the Tukey HSD/Tukey–Kramer post hoc test.

Pairs	Absolute Values of Mean Differences	Standard Error of Differences SE	Tukey HSD Q Test Statistics	*p*-Value
x1–x2	0.7507	0.05248	14.3027	7.88 × 10^−11^
x1–x3	0.01026	0.05323	0.1928	0.9991
x1–x4	0.03478	0.05248	0.6626	0.9658
x2–x3	0.7609	0.05248	14.4983	7.88 × 10^−11^
x2–x4	0.7854	0.05173	15.1839	7.88 × 10^−11^
x3–x4	0.02451	0.05248	0.4671	0.9875

**Table 8:** Comparison matrix of measurement series for absolute values of mean differences.

Seria	x2	x3	x4
x1	0.75	0.01	0.035
x2	0	0.76	0.79
x3	0.76	0	0.025

**Figure 2:**
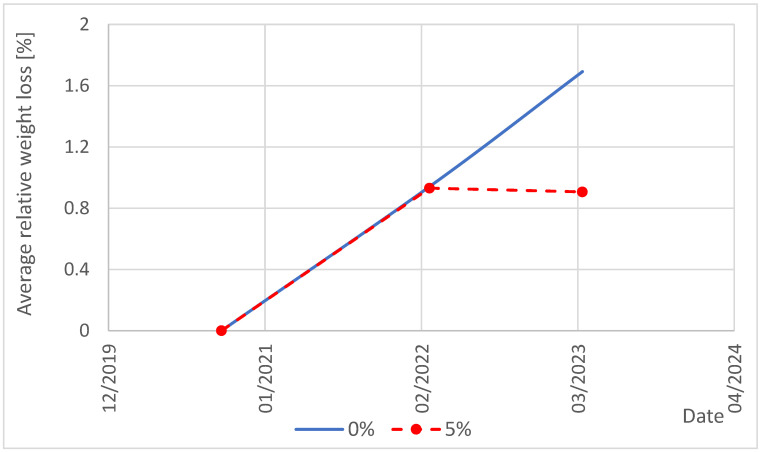
Change in relative mass loss over time for the 0% and 5% variants.

Statistically significant differences for series x1 and x2, which determine weight loss for the 0% variant, indicate that the process of weight loss increases over time. The average for series x2 is almost 80% higher than the average for series x1. The corrosion process for the 0% variant has not been stopped, as clearly illustrated in Figure 2. The lack of differences between series x1 and x3 (measurements for both variants taken in 2022) indicates that in the initial phase of corrosion, the thin outer layer was affected, and no effect of the addition of water-based resin was observed. Statistically significant differences between series x2 and x4 (measurements taken for both variants in 2023) indicate that the corrosion process does not proceed in the same way. For the 5% variant, both series x3 and x4 do not differ statistically, which indicates a slowdown in the corrosion process. The measurement in 2023 was taken after 12 months, and the mass loss is almost the same as in the initial period (the average for series x4 is slightly lower than for series x3). This process is also well illustrated in Figure 2, which shows that the average relative mass loss for the 5% variant does not change between the measurements in 2022 and 2023.

In summary, the ANOVA test showed statistically significant differences between the mean values in some series. The results of Tukey’s test specify which series differ from each other. A low *p*-value (<0.05) and a high Q-statistic indicate that these differences are significant for the interpretation of the results. Statistical tests confirmed that the addition of 5% water-soluble resin slowed down the sulfate corrosion process compared to the variant without this additive.

### 3.2. Short-Term Testing—Mass Loss and Chemical and Surface Analysis of Samples

An additional test to confirm the effectiveness of epoxy resins as a means of inhibiting sulfate corrosion involved placing samples made of standard mortar with added epoxy resin in a sulfuric acid solution with a pH of 2. These samples were identical to those used in the experiment to test the progress of carbonation. They were stored in acid for 6 months, then dried and weighed. Next, using a steel brush, the corroded layer of mortar was carefully removed, and the samples were weighed again. The difference in mass is the measure of this experiment. The results are presented in Table 9.

**Figure 3:**
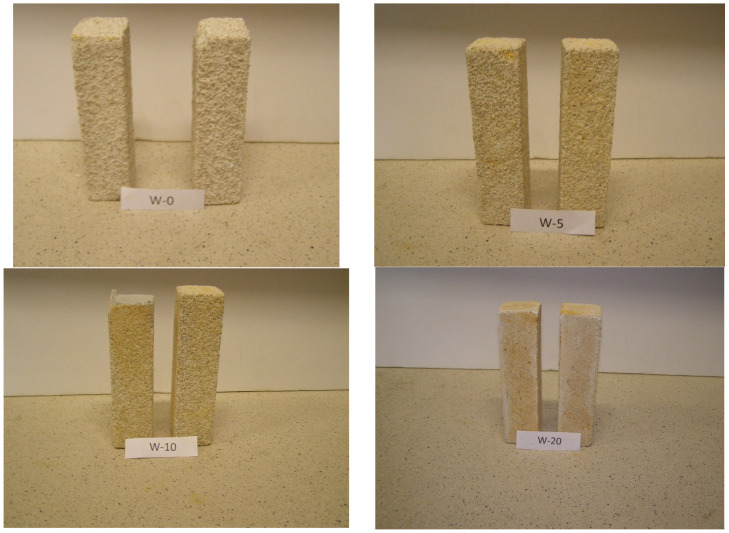
Degree of damage to the surface of the samples after stored in acid for 6 months.

An analysis of the results shown in the column presenting average values clearly shows that as the epoxy resin content in the mortar increases, so does its resistance to sulfuric acid. Samples with 20% resin content showed minimal weight loss, indicating their high resistance to chemical degradation, almost eliminating the process of sulfate corrosion. It can therefore be concluded that the use of epoxy resins as an admixture in concrete can significantly increase its durability in chemically aggressive environments. Samples with lower resin content (5% and 10%) also showed increased resistance compared to the reference sample, but not as effective as samples with 20% content. The photo below (Figure 3) illustrates the degree of damage to the surface of the samples after the experiment, clearly showing the differences in the degree of degradation depending on the epoxy resin content.

**Figure 4:**
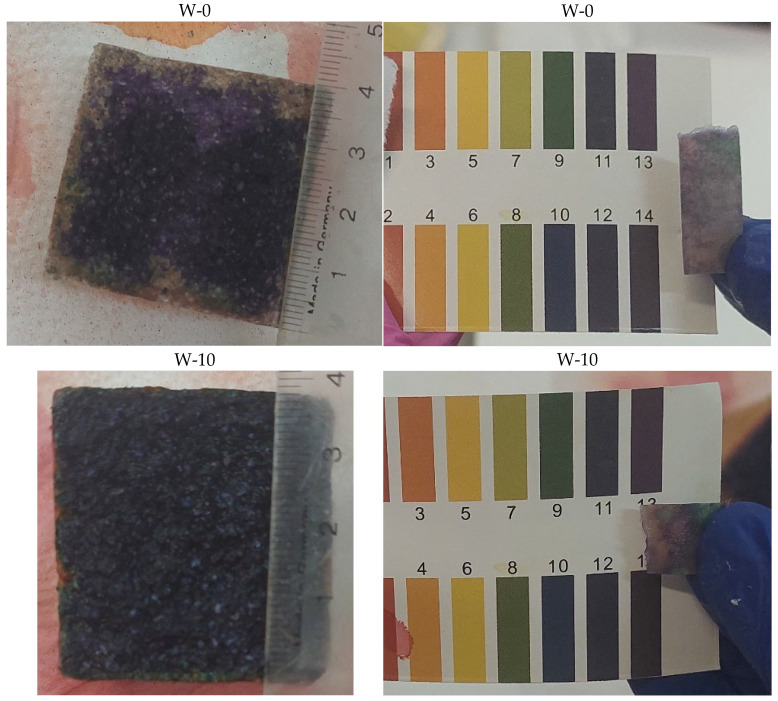
View of the samples used for the acid penetration study; the marked zones indicate the locations of pH measurements at different depths of the samples.

In order to determine the degree of acid penetration into the cementitious material, pH tests were carried out at various depths of the samples. Illustrative images of the samples are shown in Figure 4.

Based on the pH results of concrete samples (Table 10) with different resin contents (W-0, W-5, W-10, W-20) that were exposed to sulfuric acid with a pH of 2 for a period of 6 months, significant differences in their chemical resistance can be observed. In the case of sample W-0, which did not contain resin, the pH in the surface layer (up to 0.5 cm) was 5–6, which indicates significant acid penetration into the concrete structure. At greater depths (above 0.5 cm), the pH was 9, which indicates that the core of the sample remained alkaline, but also that the acid penetrated relatively deeply. In sample W-5, containing 5% resin, the zone with an acidic pH (5–6) was limited to a depth of only 0.2 cm, which means that the resin effectively increased the concrete’s resistance to acid penetration. In deeper layers, the pH was 9–11, suggesting that the alkalinity of the structure was better preserved than in the sample without resin. Sample W-10, containing 10% resin, showed even greater resistance, as the zone with a pH of 5–6 was limited to a layer 0.1 cm deep, and below this depth the pH was 13, indicating excellent protection of the alkalinity of the concrete. Sample W-20, with 20% resin content, showed the highest resistance, as the pH in the surface layer was 9, and at a depth greater than 0.1 cm, it reached a value of 13, which indicates minimal acid penetration and preservation of the strongly alkaline core of the concrete. Based on these results, it can be concluded that an increase in the resin content of concrete significantly increases its resistance to chemical degradation in an acidic environment.

### 3.3. Chemical Composition of Samples (EDS Analysis)

The use of EDS spectroscopy allowed for the examination of the elemental composition of samples after exposure to sulfuric acid.

For sample W-0 (without resin additive), EDS analysis (Table 11) showed that oxygen and calcium have the highest share, which is characteristic of concrete, whose basic components are calcium hydroxide (portlandite) and calcium carbonate (calcite). The presence of sulfur at a level of 12.06% indicates an intense reaction of sulfuric acid with concrete, resulting in the formation of gypsum (CaSO_4_·2H_2_O)—a typical product of the reaction of sulfuric acid with calcium contained in cement. The high sulfur content suggests that the sulfate corrosion process is progressing intensively, leading to weakening of the concrete structure through the formation of gypsum and ettringite (both compounds tend to expand, causing microcracks and disintegration of the concrete matrix). The silicon (Si) content of 3.87% suggests the presence of quartz (SiO_2_) or residues of incompletely hydrated cement grains. Silicon does not directly participate in the acid corrosion process, but its presence is important from the point of view of the mechanical strength of concrete.

In the case of sample W-10 (with 10% resin added), significant changes in the elemental composition were observed (Table 12). The increased carbon (C) content of 6.05% indicates the presence of epoxy resin in the concrete structure. Epoxy resin is rich in carbon, which confirms its presence and its role in protecting concrete from sulfuric acid. The carbon content comes mainly from the polymer matrix of the resin. The reduced sulfur (S) content compared to the reference sample suggests that the addition of resin limits the penetration of sulfuric acid into the concrete, leading to fewer reaction products such as gypsum and ettringite. The resin forms a protective barrier, reducing the number of pores through which aggressive sulfate ions could penetrate. The high calcium (Ca) content still suggests the presence of portlandite and calcite, but their role in protecting the concrete structure is partially reduced by the presence of the resin.

In sample W-20 (with 20% resin added), the results indicate even more favorable protective effects (Table 13). The sulfur content dropped to 8.52%, which means that the 20% resin additive protects the concrete even more effectively against sulfuric acid penetration. The decrease in sulfur content means fewer corrosion products, such as gypsum and ettringite, which translates into better durability of concrete in aggressive environments. The carbon (C) content of 6.05% confirms the presence of epoxy resin in an even higher concentration than in sample W-10, suggesting that a higher resin addition provides better protection. The increased amount of resin leads to more effective insulation of concrete from aggressive external factors. The significant silicon (Si) content of 17.92% suggests that the resin not only reduces corrosion but also changes the structure of the cement matrix. The presence of silica in such large quantities may indicate that the internal structure of the concrete remains intact, confirming the effectiveness of the protection.

EDS analysis provides key information about the processes occurring in concrete under the influence of sulfuric acid. Reference sample W-0 shows severe sulfate corrosion, which is confirmed by the high content of sulfur and chemical reaction products such as gypsum and ettringite. The use of epoxy resin in samples W-10 and W-20 effectively limited acid penetration and reduced the amount of sulfur, indicating fewer corrosion products. This interpretation is consistent with the pH penetration profiles, which show a shallower acid-affected zone with increasing resin dosage. The concurrent decrease of sulfur signal in EDS in the near-surface region supports the conclusion that resin modification reduces the ingress of aggressive species and/or slows down sulfur-related reaction product formation. At the same time, the higher carbon contribution observed in the resin-modified mixes is consistent with the presence of an organic (polymer) phase within the matrix. It should be noted that EDS provides local, semi-quantitative elemental information; therefore, these findings are presented as supportive evidence aligned with the pH profiles and mass-loss trends rather than as a direct identification of polymer distribution.

## 4. Discussion

The use of water-dilutable epoxy resins is an effective way of reducing the rate of sulfate corrosion in concrete; modified samples showed lower mass loss and better microstructural integrity than reference samples, which was interpreted as a result of reduced permeability, matrix sealing, and pore connectivity interruption, in line with observations for epoxy-modified mortars and grouts [41,42,43,44]. Mechanistically, the protective action of water-dilutable/water-soluble epoxy used as an internal admixture can be interpreted as the formation of a polymer phase within the cementitious matrix that modifies both transport and interfacial behavior. Literature on epoxy- and water-soluble epoxy-modified mortars and grouts indicates that epoxy can disperse in the fresh system and, during curing, form a continuous or semi-continuous polymer film/network within pore space and along internal surfaces, which reduces pore connectivity and blocks preferential pathways for the ingress of aggressive species [41,42]. More detailed microstructural investigations on waterborne epoxy–cement pastes report resin network formation within the hydrated matrix and changes in early hydrate development (including portlandite crystallization), supporting the concept of a dose-dependent modification of hydration products and internal morphology [45,46]. At the interfacial level, epoxy–cement paste interactions have been shown to influence failure mechanisms and the structural-to-interfacial fracture transition, which is consistent with a microcrack-bridging and bonding contribution of the polymer phase rather than only a “surface coating” effect [43,44]. Additionally, chemical characterization studies of water-soluble epoxy resin modified cement grout and broader analyses of polymer–cement interactions suggest that the polymer phase can alter the local chemistry and microstructure at phase boundaries, further supporting the proposed internal barrier and interfacial mechanisms [19,26]. Because the present work did not directly quantify pore-structure refinement or polymer spatial distribution, the above mechanism is presented as a literature-supported interpretation consistent with our observations rather than as a directly measured microstructural proof. In studies of resistance to H_2_SO_4_ and in analyses of sensitivity to test conditions, it was indicated that control of transport and shallowing of the reaction front are the main determinants of the rate of degradation, which is consistent with the reduction in mass loss observed in these samples Mechanistically, it has been emphasized that in sewage environments, where biogenic corrosion occurs through the oxidation of H_2_S to H_2_SO_4_, the effectiveness of protection is conditioned by diffusion limitation and the maintenance of an alkaline core—this effect has been confirmed by us with a shallower zone of reduced pH and less sulfur accumulation in the EDS analysis [10,47,48,49]. At the same time, it has been shown that polymer-modified materials and epoxy-based mortars can outperform surface coatings alone thanks to their barrier effect across the entire cross-section and lower susceptibility to local leaks, which is a significant application advantage in operation [40,50]. According to critical reviews and comparative studies of methods for assessing external sulfate attack, the interpretation of the results should be placed in the context of exposure conditions, which further reinforces the conclusion that transport reduction (achieved, among other things, by epoxy modification) is crucial for durability in highly chemically aggressive environments [51,52]. The results obtained indicate that the use of epoxy resins in concrete can significantly extend the durability of infrastructure exposed to a sulfate environment. Future research should consider other types of polymers and their impact on the mechanical properties of concrete. Another important issue is the long-term durability of modified concrete in real operating conditions. The results obtained may serve as a basis for further optimization of the composition of polymer concretes and their implementation in infrastructure construction.

## 5. Conclusions

- The addition of water-dilutable epoxy resin effectively limits the progression of sulfate corrosion in concrete, as confirmed by long-term and short-term tests.
- The observed reduction in mass loss and improvement in microstructure indicate the beneficial effect of the resin on the properties of concrete exposed to aggressive environments.
- Analysis of the chemical composition of the samples showed a reduction in sulfur content in samples containing resin, which proves the limited penetration of sulfate ions.
- Compared to traditional concrete protection methods, modification with epoxy resins allows the entire volume of concrete to be protected, not just the surface.
- The results suggest that the use of epoxy resins in concrete can significantly extend the durability of structures operated in environments exposed to chemical aggression.

## Figures and Tables

**Table 1:** Composition of concrete mixes per 1 m^3^.

	Ingredient						
Designation	Amount per 1 m^3^ in kg						
	Coarse Aggregate Gravel up to 16 mm	Fine Aggregate Sand up to 2 mm	Cement	Silica Fly Ash	Water	Resin—Master-Top 686	Vinapor Defoamer
W-0	1369.1	492.9	300	51.3	181.2	0	42.2
W-5	1369.1	492.9	300	51.3	159.7	36.6	42.2
W-10	1369.1	492.9	300	51.3	138.1	73.2	42.2
W-20	1369.1	492.9	300	51.3	94.9	146.3	42.2

**Table 9:** Weight loss of samples placed in sulfuric acid (pH 2) after a period of 6 months.

Variant	Sample Weights Before Cleaning [g]	Sample Weights After Cleaning [g]	Difference [g]	%	Average %
W-0	557.8	512.4	45.4	8.860	8.590
	563.7	520.4	43.3	8.321	
W-5	547.1	527.1	20	3.794	3.619
	549.6	531.3	18.3	3.444	
W-10	550.9	540.6	10.3	1.905	1.685
	526.8	519.2	7.6	1.464	
W-20	528.1	525.8	2.3	0.437	0.343
	524.4	523.1	1.3	0.249	

**Table 10:** pH summary for samples placed in sulfuric acid.

Sample	pH/Depth (cm)
W-0	h ≤ 0.5 cm—pH 5–6 h > 0.5 cm—pH 9
W-5	h ≤ 0.2 cm—pH 5–6 h > 0.2 cm—pH 9–11
W-10	h ≤ 0.1 cm—pH 5–6 h > 0.1 cm—pH 13
W-20	h ≤ 0.1 cm—pH 9 h > 0.1 cm—pH 13

**Table 11:** EDS analysis for sample W-0 soaked in sulfuric acid.

Elements	Source	% by Weight	%Atomic
C	EDS	4.36	6.88
O	EDS	64.13	76.00
Al	EDS	0.17	0.12
Si	EDS	3.87	2.61
S	EDS	12.06	7.13
Ca	EDS	15.18	7.18
Fe	EDS	0.23	0.08
Total		100	100

**Table 12:** EDS analysis for sample W-10 soaked in sulfuric acid.

Elements	Source	% by Weight	%Atomic
C	EDS	6.05	10.32
O	EDS	48.32	61.83
Mg	EDS	0.17	0.15
Al	EDS	0.69	0.53
Si	EDS	17.92	13.06
S	EDS	8.52	5.44
K	EDS	0.07	0.04
Ca	EDS	13.37	6.83
Ti	EDS	0.19	0.08
Cr	EDS	0.10	0.04
Fe	EDS	4.58	1.68
Total		100.00	100.00

**Table 13:** EDS analysis for sample W-20 soaked in sulfuric acid.

Elements	Source	% by Weight	%Atomic
C	EDS	6.05	9.6
O	EDS	57.59	68.6
Al	EDS	0.04	0.03
Si	EDS	17.92	12.16
S	EDS	8.52	5.06
Ca	EDS	8.7	4.14
Fe	EDS	1.18	0.41
Total		100.00	100.00

## Data Availability

The original contributions presented in this study are included in the article/Supplementary Material. Further inquiries can be directed to the corresponding author.

## Supplementary Materials

The following supporting information can be downloaded at: https://www.mdpi.com/article/10.3390/ma19020364/s1, Table S1: Summary of initial sample weights for laboratory weight loss tests; Table S2: Weight loss of samples immersed in sulfuric acid solution over time for the 0% variant; Table S3: Mass loss over time in samples immersed in sulfuric acid solution for the 5% variant.
