# Supplementary material for: Water-Soluble Epoxy Resins as an Innovative Method of Protecting Concrete Against Sulfate Corrosion
Source: Materials (Basel). 2026 Jan 16;19(2):364. doi: 10.3390/ma19020364 (PMC12843053; doi:10.3390/ma19020364)
Supplement: Supplementary file 1 [file materials-19-00364-s001.zip › materials-4034150-supplementary.pdf]

Raw data (initial weights) for n = 36 specimens per variant

Table S1. Summary of initial sample weights for laboratory weight loss tests.

Sample no.	0% option	5% option
1	958.81	950.82
2	830.26	821.51
3	855.48	847.38
4	1017.02	1007.29
5	1019.27	1010.80
6	845.36	838.94
7	854.94	846.26
8	938.28	933.54
9	909.44	902.80
10	902.20	896.41
11	899.80	891.44
12	939.90	929.64
13	857.46	850.44
14	1027.38	1019.02
15	912.43	904.05
16	983.47	974.22
17	879.21	870.65
18	798.21	790.66
19	983.89	972.08
20	928.73	921.49
21	911.51	903.53
22	958.39	946.37
23	982.33	973.07
24	917.74	907.57
25	997.93	989.76
26	870.88	862.36
27	893.42	882.82
28	888.64	881.48
29	944.96	937.00
30	1035.85	1024.92
31	885.70	873.89
32	929.97	920.30
33	910.76	900.41
34	979.09	962.20
35	878.05	869.86
36	867.11	853.79

Raw data for sample weight loss measurements for the 0% and 5% variants

Tables S2. Weight loss of samples immersed in sulfuric acid solution over time for the 0% variant.

Sample number	Initial weight [g]	Sample weight [g] (01-03-2022)	Weight loss [g] (01-03-2022)	Percentage weight loss [%] (01-03-2022)	Sample weight [g] (27-03-2023)	Weight loss [g] (27-03-2023)	Percentage weight loss [%] (27-03-2023)
1	958.81	950.82	7.99	0.833	932.33	18.49	1.928
2	830.26	821.51	8.75	1.054	807.07	14.44	1.739
3	855.48	847.38	8.1	0.947	832.11	15.27	1.785
4	1017.02	1007.29	9.73	0.957	994.71	12.58	1.237
5	1019.27	1010.8	8.47	0.831	998.61	12.19	1.196
6	845.36	838.94	6.42	0.759	826.89	12.05	1.425
7	854.94	846.26	8.68	1.015	829.19	17.07	1.997
8	938.28	933.54	4.74	0.505	923.1	10.44	1.113
9	909.44	902.8	6.64	0.73	885.31	17.49	1.923
10	902.2	896.41	5.79	0.642	882.25	14.16	1.569
11	899.8	891.44	8.36	0.929	878.67	12.77	1.419
12	939.9	929.64	10.26	1.092	915.55	14.09	1.499
13	857.46	850.44	7.02	0.819	829.19	21.25	2.478
14	1027.38	1019.02	8.36	0.814	998.76	20.26	1.972
15	912.43	904.05	8.38	0.918	890.37	13.68	1.499
16	983.47	974.22	9.25	0.941	958.78	15.44	1.57
17	879.21	870.65	8.56	0.974	854.4	16.25	1.848
18	798.21	790.66	7.55	0.946	774.32	16.34	2.047
19	983.89	972.08	11.81	1.2	959.05	13.03	1.324
20	928.73	921.49	7.24	0.78	908.35	13.14	1.415
21	911.51	903.53	7.98	0.875	886.24	17.29	1.897
22	958.39	946.37	12.02	1.254	929.67	16.70	1.743
23	982.33	973.07	9.26	0.943	955.49	17.58	1.790
24	917.74	907.57	10.17	1.108	892.32	15.25	1.662
25	997.93	989.76	8.17	0.819	966.83	22.93	2.298
26	870.88	862.36	8.52	0.978	846.88	15.48	1.778
27	893.42	882.82	10.6	1.186	869.52	13.3	1.489
28	888.64	881.48	7.16	0.806	866.0	15.48	1.742
29	944.96	937.0	7.96	0.842	929.33	7.67	0.812
30	1035.85	1024.92	10.93	1.055	1014.84	10.08	0.973
31	885.7	873.89	11.81	1.333	859.24	14.65	1.654
32	929.97	920.3	9.67	1.04	900.44	19.86	2.136
33	910.76	900.41	10.35	1.136	881.59	18.82	2.066
34	979.09	962.2	16.89	1.725	943.83	18.37	1.876
35	878.05	869.86	8.19	0.933	854.2	15.66	1.783
36	867.11	853.79	13.32	1.536	834.56	19.23	2.218

Table S3. Mass loss over time in samples immersed in sulfuric acid solution for the 5% variant

Sample number	Initial weight [g]	Sample weight [g] (01-03-2022)	Weight loss [g] (01-03-2022)	Percentage weight loss [%] (01-03-2022)	Sample weight [g] (27-03-2023)	Weight loss [g] (27-03-2023)	Percentage weight loss [%] (27-03-2023)
1	965.92	956.37	9.55	0.989	942.25	14.12	1.462
2	815.79	805.83	9.96	1.221	791.82	14.01	1.717
3	905.78	898.55	7.23	0.798	888.55	10.0	1.104
4	905.89	898.96	6.93	0.765	883.47	15.49	1.71
5	913.83	905.34	8.49	0.929	894.65	10.69	1.17
6	983.92	975.12	8.8	0.894	960.38	14.74	1.498
7	935.12	926.45	8.67	0.927	913.7	12.75	1.363
8	901.08	892.09	8.99	0.998	878.63	13.46	1.494
9	880.2	872.71	7.49	0.851	858.92	13.79	1.567
10	930.3	921.95	8.35	0.898	909.96	11.99	1.289
11	951.7	943.16	8.54	0.897	934.95	8.21	0.863
12	896.96	887.91	9.05	1.009	877.93	9.98	1.113
13	981.34	973.15	8.19	0.835	963.11	10.04	1.023
14	952.96	944.32	8.64	0.907	934.94	9.38	0.984
15	943.17	932.87	10.3	1.092	924.03	8.84	0.937
16	892.31	886.58	5.73	0.642	873.15	13.43	1.505
17	887.38	878.35	9.03	1.018	869.54	8.81	0.993
18	950.88	942.3	8.58	0.902	933.43	8.87	0.933
19	878.42	869.57	8.85	1.007	863.54	6.03	0.686
20	887.88	880.68	7.2	0.811	874.83	5.85	0.659
21	887.16	878.68	8.48	0.956	874.0	4.68	0.528
22	911.96	904.74	7.22	0.792	898.57	6.17	0.677
23	853.67	846.9	6.77	0.793	843.43	3.47	0.406
24	981.68	974.75	6.93	0.706	967.6	7.15	0.728
25	913.09	903.54	9.55	1.046	898.18	5.36	0.587
26	934.51	923.72	10.79	1.155	919.63	4.09	0.438
27	858.26	849.06	9.2	1.072	842.87	6.19	0.721
28	927.25	917.34	9.91	1.069	914.66	2.68	0.289
29	863.35	857.71	5.64	0.653	855.46	2.25	0.261
30	883.63	876.5	7.13	0.807	874.7	1.8	0.204
31	840.82	833.22	7.6	0.904	829.1	4.12	0.49
32	1008.93	998.11	10.82	1.072	994.2	3.91	0.388
33	888.05	880.39	7.66	0.863	872.08	8.31	0.936
34	894.67	878.98	15.69	1.754	872.25	6.73	0.752
35	964.15	950.97	13.18	1.367	946.13	4.84	0.502
36	880.93	867.96	12.97	1.472	862.26	5.7	0.647
